# The correlation between *Demodex* infestation and meibomian gland dysfunction at different ages

**DOI:** 10.1186/s12886-022-02610-9

**Published:** 2022-10-01

**Authors:** Xiaowen Sun, Zhanglin Liu, Shengshu Sun, Shaozhen Zhao, Xiaomin Zhang, Yue Huang

**Affiliations:** 1 Tianjin Key Laboratory of Retinal Functions and Diseases, Tianjin Branch of National Clinical Research Center for Ocular Disease, Eye Institute and School of Optometry, Tianjin Medical University Eye Hospital, Tianjin, 300384 China; 2 Ophthalmology Department of People’ Hospital of Rizhao, Rizhao, 276800 Shandong China

**Keywords:** *Demodex*, Meibomian gland dysfunction, Age

## Abstract

**Background:**

This study aimed to explore the associations between *Demodex* infestation and the ocular surface characteristics of meibomian gland dysfunction (MGD) in different age groups, to further understand the effect of *Demodex* on MGD.

**Methods:**

A total of 202 consecutive MGD patients aged 18 to 70 years were randomly recruited. All patients were divided into two groups based on their age: young patients (18–40 years) and elderly patients (41–70 years). The main observations were the different relationship between *Demodex* infestation and ocular surface and meibomian gland (MG) parameters in two age groups. We also compared ocular surface and MG parameters between the young and the elderly groups. *Demodex* infestation was diagnosed based on expert consensus in China.

**Results:**

Our results indicated significant differences among young *Demodex*-positive, suspicious-positive, and negative patients in MG dropout (*P* = 0.000), plugging of MG orifices (*P* = 0.000), lid margin abnormality (*P* = 0.000), and meibum quality (*P* = 0.000). In elderly patients, there were significant differences among the *Demodex*-positive, suspicious-positive, and negative groups in terms of ocular surface disease index (OSDI) (*P* = 0.037), fluorescein tear film break-up time (FBUT) (*P* = 0.002), corneal fluorescein staining (CFS) (*P* = 0.036), MG dropout (*P* = 0.000), plugging of MG orifices (*P* = 0.008), lid margin abnormality (*P* = 0.000), and MG expression (*P* = 0.037). The mean number of mites in elderly *Demodex*-positive patients (10.64 ± 7.50) was greater than that of in young patients (7.60 ± 4.71) (*P* = 0.014). MG dropout *(P* = 0.000), plugging of MG orifices (*P* = 0.006), lid margin abnormality (*P* = 0.000), MG expression(*P* = 0.001), and meibum quality (*P* = 0.032) were more severe in elderly *Demodex*-positive patients. Additionally, FBUT (*P* = 0.005) was lower and tear film lipid layer thickness (LLT) (*P* = 0.001) was higher in the elderly.

**Conclusion:**

The effect of *Demodex* infestation on the ocular surface and MG parameters of MGD was different in patients of different ages. It is necessary to pay more attention to the diagnosis and treatment of *Demodex* infestation in MGD.

## Background

Dry eye disease (DED) is one of the most common ocular surface disorders. It is caused by multiple factors and has the potential to impact the patient's quality of life. It is characterised by unstable tear film, which causes a variety of symptoms and can potentially be accompanied by ocular surface damage [1, 2]. Meibomian gland dysfunction (MGD) is the major cause of evaporative DED and is characterized by obstruction of the MG terminal ducts and/or changes in their glandular secretion [3]. MGD is a type of posterior blepharitis characterised by inflammation behind the grey line of the eyelid margin, which impairs the stability of the tear film, leading to irritation and visual disturbances [4]. Epidemiological studies around the world have reported the prevalence of MGD as ranging from 20 to 70%, with incidence increasing with age [5–7]. MGD is influenced by various factors, including age, sex, environmental stress, hormone levels, medications, dietary intake, and contact lens wear. *Demodex* infestation is also considered a risk factor for MGD [8].

*Demodex folliculorum (D. folliculorum)* and *Demodex brevis (D. brevis)* are two separate mites that are common obligate parasites in humans; the former mainly exists in clusters in the eyelash follicles, whereas the latter resides deep in the sebaceous glands and MGs [9]. Since animal models of ocular *Demodex* infestation have not been successfully established, the pathogenesis of *Demodex* infestation remains controversial, with plausible explanations including causing direct injury, acting as a bacterial transporter, and inducing allergy [10]. The prevalence of *Demodex* infestation has been shown to increase with age and was reported in 84% of people over the age of 60 and 100% of people over 70 years old [11]. Ocular *Demodex* is associated with various systemic impaired immunity and dermatology, such as obesity, malignancy, diabetes mellitus, acquired immunodeficiency syndrome, acne vulgaris and rosacea [12]. *Demodex* infestation has also been implicated as a potential cause of ocular diseases such as eyelash loss, abnormal eyelash alignment, blepharitis, blepharoconjunctivitis, pterygium, MGD, keratitis, and eyelid basal cell carcinoma [11]. Other studies, however, have indicated that *Demodex* was non-pathogenic because it was asymptomatic in some humans [13]. Immunocompetent paediatric patients were also found to have ocular *Demodex* infestation [14].

Research between *Demodex* presence and parameters of ocular surface damage is still inconsistent. A study of patients newly diagnosed with DED showed that lower Schirmer test and higher ocular surface disease index (OSDI) scores were significantly associated with *Demodex* infestation [13]. However, Rabensteiner et al. reported higher OSDI values in *Demodex* negative patients than in positive patients. This study also reported lower MG secretion quality and higher Marx line (ML) score in *Demodex* positive patients, but *Demodex* infestation was not significantly correlated with MG expression, MG dropout, or MGD diagnosis [15]. However, another study reported that *Demodex* could induce microstructural changes in MGs and aggravate MGD [16].

We speculated that these varying results may be attributed to factors such as differences in subjects, age, detection technology, and diagnostic criteria for *Demodex* infestation. Most previous studies focused on the effect of *Demodex* infestation in anterior blepharitis, but fewer on the effect in MGD, and their relationship has been debatable. In this study, we explored *Demodex* infestation in MGD. Our main purpose was to investigate relationships between *Demodex* presence and ocular surface characteristics of MGD, and further, to observe the role of age on ocular surface changes in *Demodex* infestation and better understand their relationships. This study will provide a theoretical basis for clinical diagnosis and therapy for MGD with *Demodex* infestation.

## Methods

### Subjects

This cross-sectional study was conducted at Tianjin Medical University Eye Hospital Dry Eye outpatient clinic. A total of 202 consecutive MGD patients aged 18 to 70 years were recruited randomly from April 2021 to January 2022. The inclusion criteria were as follows: patients combined with ocular symptoms and diagnosed with MGD for the first time who had not received any type of dry eye treatment before recruitment. MGD is defined as a chronic, diffuse abnormality of the meibomian glands, characterized by terminal duct obstruction and/or qualitative/quantitative changes in the glandular secretion [3]. The exclusion criteria were as follows: patients suffering from anterior blepharitis; patients with a history of ocular surgery, trauma, or chemical burns within the past three months; patients who received eye treatment with systemic antibiotics or topical eye drops within six months; patients suffering from systemic or local infectious diseases; and patients receiving therapy with steroid hormones and immunosuppressive drugs. The protocol of this study strictly followed the guidelines of the Declaration of Helsinki and was approved by the Ethics Committee of Tianjin Medical University Eye Hospital (2021KY[L]-03). The protocol was fully explained to all subjects who provided written informed consent prior to the beginning of the study.

All patients were divided into two groups based on their age: young patients(18–40 years) and elderly patients(41–70 years). The main observations were the different relationships between ocular *Demodex* infestation and ocular surface characteristics in two different age groups.

Ocular surface characteristics were measured using the assessments in the following order of least invasive to most invasive: OSDI questionnaire, lid margin abnormality, plugging of MG orifices, lipid layer thickness(LLT), fluorescein tear film break-up time (FBUT), corneal fluorescein staining(CFS), MG dropout, meibum expression, meibum quality, and Schirmer I test. The OSDI questionnaire was completed by one ophthalmologist for each patient, and each examination was assessed by the same experienced ophthalmologist.

#### Diagnosis of ocular Demodex infestation

Three eyelashes from each of the eyelids, along the nasal, centre, and temporal side, were in turn epilated under a slit-lamp microscope. A total of 12 eyelashes, specially selected with cylindrical dandruff (CD), were removed and placed on a glass slide. A coverslip was placed on the lashes and two drops of normal saline solution were slowly pipetted at the edge of the coverslip. *Demodex-*mites were examined under a microscope at 10 × and 40 × magnifications after 20 min. The total number of mites was summed from the two eyes.

The diagnosis of ocular *Demodex* infestation was based on the expert consensus on the diagnosis and treatment of *Demodex* blepharitis in China as follows [17]: (1) *Demodex* were counted in all stages. (2) *Demodex* counts in adult patients were more than or equal to 3 per 3 eyelashes in any of the four eyelids. (3) A number less than the above standard was considered suspicious-positive. Combination with clinical manifestations was essential. Ocular parameters were observed among *Demodex* positive, suspicious-positive, and negative groups in each age group.

### Ocular surface parameters

(1)OSDI: The OSDI questionnaire was used to assess dry eye symptoms within the past two weeks. The OSDI questionnaire consisted of 12 questions with a total score ranging from 0 to 100. A higher total score indicated greater severity of dry eye symptoms [18]. (2)Corneal fluorescein staining: The cornea was separated into four quadrants and each quadrant was scored individually on a scale of 0–3, with a maximum total score of 12 [19]. (3) Fluorescein tear film break-up time: FBUT was assessed after CFS measurements and blinking three times under a cobalt blue light with the averages of three values recorded. (4)Schirmer test I (SIT)*:* SIT was assessed without topical anaesthesia for 5 min. (5)Tear film lipid layer thickness: LLT was detected with a LipiView Ocular Surface Interferometer (Johnson & Johnson, New Brunswick, NJ). LipiView can directly measure the LLT through images. Since the LLT value cannot be estimated accurately when it exceeds 100 nm, we recorded the LLT value as 100 nm when LLT > 100 nm [20].

### MD parameters

(1)MG dropout: MG dropout was assessed through the Keratograph 5 M (Oculus, Arlington, WA), which captured MG images using infrared light. MG dropout was scored from 0 to 3 for each eyelid ((0 = no loss of MG; 1 = loss of MG < 1/3 area; 2 = loss of MG 1/3–2/3 area; 3 = loss of MG > 2/3 area) [21]. (2)Plugging of MG orifices: Plugging of MG orifices was scored from 0 to 3 (0 = no plugging of orifices; 1 = plugging of fewer than 3 orifices; 2 = plugging of 3 or more orifices with a distribution of less than half of the full length of the lid; 3 = plugging of 3 or more orifices with a distribution of half or more of the full length of the lid) [22]. (3)Lid margin abnormality: Lid margin abnormalities, including four parameters (irregular lid margin, vascular engorgement, plugged MG orifices, and anterior or posterior replacement of mucocutaneous junction), were scored from 0 to 4 (0 = absent or 1 = present) based on the number of parameters present with a total score of 0–4 in each eyelid [23]. (4)MG expression: Each part of five MGs in the nasal, middle, and temporal regions of the eyelid were evaluated on a scale of 0–3 for a total score of 0–9 (0 = expression from all 5 glands; 1 = expression from 3 to 4 glands; 2 = expression from 1 to 2 glands; 3 = no expression) [24]. (5)Meibum quality: Meibum quality was evaluated from eight glands at the centre of the eyelid using a score of 0–3 for each gland with a total score of 0–24 (0 = clear; 1 = cloudy; 2 = cloudy with granular debris; 3 = thick, like toothpaste) [25]. The total score of each eye from the above parameters was summed together from the upper and lower eyelids.

### Statistical analysis

All statistical calculations of the data were performed using SPSS software (version 23.0; IBM, Armonk, NY), and *P* < 0.05 was considered statistically significant. All right eyes were selected for statistical analysis.

Descriptive statistics were presented as mean ± standard deviation (SD) for continuous variables and percentages for categorical variables. The Kolmogorov–Smirnov (K-S) test was used to assess whether the continuous variables were normally distributed. Continuous variables with normal distribution were analysed by independent T-test and one-way analysis of variance (ANOVA) and the Mann–Whitney U and Kruskal–Wallis H tests were used to analyse non-normally distributed data. Categorical parameters were assessed with Pearson’s χ^2^ test. Spearman correlation analysis and linear regression analysis was used to evaluate the correlations between *Demodex* count and ocular parameters.

## Results

### General analysis

Among the 202 enrolled patients (69 males, 133 females), the prevalence of *Demodex* infestation was 51.5% (*n* = 104). 41(20.3%) patients were classified as *Demodex* suspicious-positive and 57 (28.2%) as *Demodex* negative. There were significant differences in age among patients who were *Demodex* positive (43.73 ± 15.68 years), suspicious-positive (40.71 ± 17.47 years), and negative (35.60 ± 13.14 years) (*P* = 0.007). The average number of *Demodex* mites per patient was 5.36 ± 6.20 per 12 eyelashes (Table 1).

**Table 1:** Demographics of all *Demodex* positive, suspicious-positive and negative patients

	Positive	Suspicious positive	Negative	*P* value
Total, n (%)	104 (51.5%)	41 (20.3%)	57 (28.2%)	0.000*
Mean age	43.73 ± 15.68	40.71 ± 17.47	35.60 ± 13.14	0.007^
Sex; male (n)	38	11	20	0.532*
female(n)	66	30	37	

^*^P-value determined by Pearson’sχ^2^ test

^ *P*-value determined by Kruskal- Wallis H test

Significant *p*-values (*P* < 0.05) are in bold

The young group comprised 109 individuals (43 males, 66 females), and the elderly group included 93 individuals (26 males, 67 females). The two different age groups were matched in sex distribution (*P* = 0.077). The prevalence of ocular *Demodex* infestation in elderly patients (60.2%) was higher than that in young patients (44.0%) (*P* = 0.042).

### Demodex infestation in young patients

Table 2 shows the analysis of all parameters for the *Demodex* positive, suspicious-positive, and negative groups in young patients. There were no significant differences among the three groups in age (*P* = 0.352) or sex (*P* = 0.135). There were no significant differences in OSDI, CFS, FBUT, SIT, LLT, and MG expression among the three groups (*P* > 0.05). However, there were significant differences in MG dropout (*P* = 0.000), plugging of MG orifices (*P* = 0.000), lid margin abnormality (*P* = 0.000), and meibum quality (*P* = 0.000).

**Table 2:** Comparison of ocular surface and MG parameters among three groups in young patients

	Positive	Suspicious positive	Negative	*P* value
OSDI	23.11 ± 11.48	22.45 ± 8.97	23.64 ± 11.51	0.923*
FBUT	4.85 ± 2.04	5.36 ± 2.63	5.49 ± 1.88	0.243
CFS	0.98 ± 1.60	0.77 ± 1.07	0.63 ± 1.15	0.058
SIT(mm)	10.66 ± 7.87	9.38 ± 5.70	11.08 ± 7.87	0.844*
LLT(nm)	62.68 ± 23.37	64.35 ± 26.52	74.89 ± 21.01	0.054
MG dropout	2.31 ± 1.52	1.52 ± 1.12	1.05 ± 1.14	**0.000**
Plugging of MG orifices	4.60 ± 1.21	3.44 ± 1.10	3.29 ± 1.58	**0.000**
Lid margin abnormality	3.36 ± 1.03	2.27 ± 1.12	2.00 ± 1.09	**0.000**
MG expression	5.06 ± 3.35	3.77 ± 2.58	3.55 ± 3.25	0.059
Meibum quality	12.81 ± 5.32	8.77 ± 4.39	7.89 ± 4.44	0.000*

*MG* Meibomian gland, *OSDI* Ocular surface disease index, *FBUT* Fluorescein tear film break-up time, *CFS* Corneal fluorescein staining, *SIT* Schirmer test I, *LLT* Lipid layer thickness

*P*-value determined by Kruskal–Wallis H test; * indicates analysis of variance

Significant *P*-values (*P* < 0.05) are in bold

When comparing the two groups, a significant difference was only seen between the *Demodex* positive and negative groups in MG dropout (*P* = 0.000), with more severe MG dropout seen in the positive group. There were significant differences between the positive and suspicious-positive groups, and between the positive and negative group, in plugging of MG orifices (*P* = 0.011, *P* = 0.001, respectively), lid margin abnormality (*P* = 0.002, *P* = 0.000, respectively) and meibum quality (*P* = 0.002, *P* = 0.000, respectively), where the score in the positive group was higher than the other two groups.

### Demodex infestation in elderly patients

Table 3 shows the analysis of all parameters for the *Demodex* positive, suspicious-positive, and negative groups in elderly patients. Among the three groups, no significant differences were also observed in age (*P* = 0.106) or sex (*P* = 0.297). There were no significant differences in SIT, LLT, or meibum quality(*P* > 0.05). However, our analysis revealed significant differences in OSDI (*P* = 0.037), FBUT (*P* = 0.002), CFS (*P* = 0.036), MG dropout (*P* = 0.000), plugging of MG orifices (*P* = 0.008), lid margin abnormality (*P* = 0.000), and MG expression (*P* = 0.037).

**Table 3:** Comparison of ocular surface and MG parameters among three groups in elderly patients

	Positive	Suspicious positive	Negative	*P* value
OSDI	25.99 ± 11.13	20.52 ± 8.31	20.27 ± 7.98	0.037*
FBUT	3.77 ± 1.73	4.30 ± 1.80	5.37 ± 1.70	**0.002**
CFS	1.32 ± 1.88	0.61 ± 0.92	0.32 ± 0.48	**0.036**
SIT(mm)	8.72 ± 6.23	10.22 ± 6.98	9.18 ± 7.37	0.820*
LLT(nm)	79.76 ± 22.03	78.17 ± 21.36	75.22 ± 20.19	0.663
MG dropout	3.35 ± 1.29	2.22 ± 1.21	1.58 ± 1.26	**0.000**
Plugging of MG orifices	5.19 ± 1.19	4.41 ± 1.81	4.06 ± 1.47	**0.008**
Lid margin abnormality	4.32 ± 1.42	3.67 ± 1.61	2.68 ± 1.29	**0.000**
MG expression	7.45 ± 3.57	6.72 ± 4.40	4.95 ± 2.72	0.037*
Meibum quality	14.89 ± 4.21	12.61 ± 4.79	12.58 ± 5.02	0.059*

*MG* Meibomian gland, *OSDI* Ocular surface disease index, *FBUT* Fluorescein tear film break-up time, *CFS* Corneal fluorescein staining, *SIT* Schirmer test I, *LLT* Lipid layer thickness

*P*-value determined by Kruskal–Wallis H test; * indicates analysis of variance

Significant *P*-values (*P* < 0.05) are in bold

When comparing the two groups, significant differences were only seen between *Demodex* positive and negative groups in CFS (*P* = 0.048), FBUT (*P* = 0.001), plugging of MG orifices (*P* = 0.008), lid margin abnormality (*P* = 0.000), and MG expression (*P* = 0.010). Patients who were *Demodex* positive had significantly lower FBUT and higher CFS, plugging of MG orifices, lid margin abnormality, and MG expression compared to the negative group. Our results also showed significant differences between the positive and suspicious-positive groups as well as the positive and negative groups in OSDI (*P* = 0.048, *P* = 0.035, respectively) and MG dropout (*P* = 0.005, *P* = 0.000, respectively), with the positive group having a higher score than the other groups.

### Age group comparison of all Demodex-positive patients

Among the *Demodex* positive patients, 48 cases (24 male, 24 female) were in the young group, and 56 cases (14 male, 42 female) were in the elderly group. Significant differences were observed in the sex distribution between the two age groups (*P* = 0.008). The mean number of *Demodex* mites in elderly patients (10.64 ± 7.50) was greater than that in young patients (7.60 ± 4.71) (*P* = 0.014).

Compared to young patients, MG dropout (*P* = 0.000), plugging of MG orifices (*P* = 0.006), lid margin abnormality (*P* = 0.000), MG expression (*P* = 0.001), and meibum quality (*P* = 0.032) were more severe, LLT(*P* = 0.001) was higher, and FBUT(*P* = 0.005) was lower in the elderly group. The OSDI and CFS were higher and SIT lower in the elderly group, but these differences were not significant (*P* > 0.05) (Table 4).

**Table 4:** Comparison of ocular surface and MG parameters between the young and elderly *Demodex* positive patients

	Young	Elderly	*P* value
Mean *Demodex* counts	7.60 ± 4.71	10.64 ± 7.50	**0.014**
Sex; male (n)	24	14	**0.008**^
female	24	42	
OSDI	23.11 ± 11.48	25.99 ± 11.13	0.207*
FBUT	4.85 ± 2.04	3.77 ± 1.73	**0.005**
CFS	0.98 ± 1.60	1.32 ± 1.88	0.182
SIT(mm)	10.66 ± 7.87	8.72 ± 6.23	0.304
LLT(nm)	62.68 ± 23.37	79.76 ± 22.03	0.001*
MG dropout	2.31 ± 1.52	3.35 ± 1.29	**0.000**
Plugging of MG orifices	4.60 ± 1.21	5.19 ± 1.19	**0.006**
Lid marginabnormality	3.36 ± 1.03	4.32 ± 1.42	**0.000**
MG expression	5.06 ± 3.35	7.45 ± 3.57	0.001*
Meibum quality	12.81 ± 5.32	14.89 ± 4.21	0.032*

*MG* Meibomian gland, *OSDI* Ocular surface disease index, *FBUT* Fluorescein tear film break-up time, *CFS* Corneal fluorescein staining, *SIT* Schirmer test I, *LLT* Lipid layer thickness

*P*-value determined by Mann–Whitney U test; ^ indicates Pearson’sχ^2^ test, * indicates independent t-test

Significant *P*-values (*P* < 0.05) are in bold

### Correlation between Demodex count and parameters in Demodex positive patients

In the young *Demodex*-positive patients, *Demodex* count was significantly correlated with lid margin abnormality (*P* = 0.049) and meibum quality (*P* = 0.038). No significant correlation was noted between *Demodex* count and other parameters(*P* > 0.05) (Table 5). In linear regression analysis, the more *Demodex* count, the lid margin abnormality was more serious(*R*^2^ = 0.209, *P* < 0.001) and the meibum quality was poorer(*R*^2^ = 0.155, *P* = 0.006).

**Table 5:** Correlation analysis between demodex count and parameters in demodex positive patients

	Young		Elderly	
	R	*P* value	R	*P* value
Age	-0.184	0.209	0.164	0.228
OSDI	0.136	0.336	0.120	0.386
FBUT	0.226	0.140	-0.192	0.172
CFS	0.139	0.351	0.099	0.466
SIT(mm)	0.209	0.173	0.012	0.931
LLT(nm)	0.214	0.149	-0.057	0.682
MG dropout	0.113	0.446	0.106	0.442
Plugging of MG orifices	0.069	0.664	0.446	**0.001**
Lid margin abnormality	0.289	**0.049**	0.193	0.155
MG expression	-0.046	0.759	0.182	0.179
Meibum quality	0.303	**0.038**	0.102	0.456

*MG* Meibomian gland, *OSDI* Ocular surface disease index, *FBUT* Fluorescein tear film break-up time, *CFS* Corneal fluorescein staining, *SIT* Schirmer test I, *LLT* Lipid layer thickness

*P*-value determined by Spearman correlation analysis

Significant *p* values *P* < 0.05 are in bold

In the elderly *Demodex*-positive patients, *Demodex* count was significantly correlated with plugging of MG orifices (*P* = 0.001). No significant correlation was found between *Demodex* count and other parameters (*P* > 0.05) (Table 5). In linear regression analysis, the more *Demodex* count, the plugging of MG orifices was more worse(*R*^2^ = 0. 105, *P* = 0.019).

## Discussion

In this study, we demonstrated the correlation between *Demodex* presence and ocular characteristics of MGD. Due to the high incidence of *Demodex* infestation in the elderly, most ocular *Demodex* studies have focused on elderly subjects. Our study investigated different influences of *Demodex* on the ocular surface and MG parameters between young and elderly MGD patients. An Austrian study on patients with symptoms of ocular discomfort had a *Demodex* prevalence of 40.2%, and the patients with *Demodex* infestation were significantly older than the *Demodex* negative patients [15]. Sędzikowska et al. showed that the older the patient, the greater the likelihood of *Demodex* infestation by applying logistic regression analysis [26]. In our study, we found that the prevalence of ocular *Demodex* in elderly patients (60.2%) was higher than the young patients (44.0%) (*P* = 0.042). Among all our enrolled patients, the mean age of the *Demodex*-positive group was older than the suspicious-positive and negative groups (*P* = 0.007), which corresponded with previous findings. This may be due to poor immune systems and declining healthy hygiene habits in elderly patients.

In our study, the average number of *Demodex* mites per patient was 5.36 ± 6.20 per 12 eyelashes. We found that the mean *Demodex* counts in elderly patients (10.64 ± 7.50 per 12 eyelashes) were greater than that of young patients (7.60 ± 4.71 per 12 eyelashes) (*P* = 0.014). Our data were consistent with that of Wesolowska et al. who found that the number of *Demodex* mites increased with age [27]. However, Li et al. showed that *Demodex* counts were comparable in young and elderly patients, while young patients had higher *D. brevis* counts and elderly patients had higher *D. folliculorum* counts [28]. It is possible that the differences in research subjects and age classification criteria among these studies may be the reason for their different results.

In young patients, *Demodex* infestation was not associated with OSDI score, while OSDI scores in elderly patients were higher in *Demodex*-positive patients than in those considered suspicious-positive and negative (*P* < 0.05). Therefore, there was no significant relationship between *Demodex* infestation and dry eye symptoms in young patients, but there was in elderly patients. The influence of *Demodex* infestation on dry eye symptoms was more serious in older patients. Ayyildiz et al. also showed a significant relationship between the OSDI score and *Demodex* infestation in individuals first diagnosed with DED aged 40 to 68 years [13]. Lee et al. found that the number of *Demodex* mites was proportional to the OSDI score and the severity of ocular discomfort [29]. However, our results showed no correlation between *Demodex* count and OSDI score in *Demodex* positive patients. Therefore, we believe that an increase in *Demodex* mites after diagnosed infestation is not associated with an increase in the frequency of severe dry eye symptoms in the elderly. Interestingly, a few individuals had no symptoms with more *Demodex* counts and severe signs. This may be due to the significantly reduced density of corneal nerves in the presence of severe *Demodex* infestation, which leads to corneal hypoesthesia [30].

In elderly *Demodex*-positive patients, CFS was more serious and FBUT was lower compared to negative patients. Sędzikowska et al. showed that, in a study of patients without visible eyelid or eye surface disorders, the first and mean BUT in the *Demodex*-infested group was shorter than that in non-infected individuals [31]. Our results indicate a significant correlation between *Demodex* infestation and lower tear film stability and corneal epithelial injury in elderly patients. Zhang et al. showed that *Demodex* may impair the barrier function of the corneal epithelium by activating the IL-17/MMP-9 signalling pathway [25]. Further, Cheng et al. suggested that microstructural changes of MGs were positively related to *Demodex* infestation and proportional to the number of mites [16]. These consequences were associated with the tear film stability, followed by damage to the ocular surface epithelium [31]. Some studies have found correlation between *Demodex* infestation and imbalance in the bacterial microbiota in the conjunctival sac which can affect the functioning of the ocular surface [32, 33]. Therefore, we considered that, in elderly patients, the influence of *Demodex* infestation on ocular surface damage may result in more severe dry eye symptoms in *Demodex*-positive patients. These findings suggest that the elimination of *Demodex* mites could alleviate dry eye symptoms in the elderly. In young patients, however, no association was found between *Demodex* infestation and CFS and FBUT. This may be because the young patients were in the early stages of *Demodex* infestation, with it lasting only a short duration, and the number of mites was small.

Previous studies reported that *D. brevis* was often found in young patients and significantly correlated with corneal involvement [28, 34]; however, this was not consistent with our results. This may be because *D. folliculorum* and *D. brevis* were not distinguished from each other, and patients with keratitis were excluded from our research. Because the identification of *D.brevis* is difficult, it is often confused with the shorter *D. folliculorum* [15]. Thus, the role of *D. brevis* should be considered in future *Demodex*-associated studies.

Our results showed that *Demodex* infestation in young patients was not associated with MG expression, indicating that *Demodex* has no obvious impact on MG expression or function in young patients. In the elderly, however, there were significant differences between *Demodex* positive and negative groups in MG expression and no differences were found between any other groups. Another study hypothesised that *Demodex* may become host pathogens if their counts exceed a critical level [35]. Considering that the number of *Demodex* mites in young patients was small and the speculated effect of *Demodex* on MG expression was significant when the number of *Demodex* reached a certain level, young MGD patients with *Demodex* infestation may require not only *Demodex* eradication but also MG physical therapy.

In young patients, the relationship between *Demodex* infestation and meibum quality was significant. The meibum quality was worse in the *Demodex*-positive group compared to the suspicious-positive and negative groups. A study on young adults (18–40 years) with and without MGD showed that *Demodex* had a remarkable impact on meibum composition, with significant changes observed in the levels of (O-acyl)-ω-hydroxy fatty acids in patients with *Demodex* infestation [7]. Furthermore, meibum secretion was more active in young individuals [28], so the changes in lipid composition may be more complicated in these patients. Therefore, *Demodex* infestation had an obvious impact on the meibum quality of the young patients. Future studies should investigate the relationship between the changes in lipid composition and clinical features. However, no significant difference was observed between *Demodex* infestation and meibum quality in the elderly. We considered that age and some other obscure factors may have a greater impact on the meibum quality of elderly patients.

Significant differences were found between *Demodex* infestation and MG dropout, as well as plugging of MG orifices and lid margin abnormality in both young and elderly patients. *Demodex* could mechanically block the orifices of MGs and hair follicles, resulting in epithelial hyperplasia and hyperkeratosis. *Demodex* could also transmit concomitant bacteria such as *Streptococci*, *Staphylococci*, and *Bacillus oleronius,* and bacterial antigens could trigger inflammatory responses. Proteins and debris from *Demodex* could induce immune responses [11, 16, 36]. The above possible mechanism may be associated with the plugging of MG orifices, MG dropout, and lid margin abnormality. Therefore, the influence of *Demodex* on MG may appear before ocular surface signs.

There were no significant differences between *Demodex* infestation and SIT in both young and elderly patients. Mizuno et al. and Rabensteiner et al. also demonstrated no association between *Demodex* presence and SIT. It was possible that *Demodex* influenced MGs, causing tear film instability, but did not influence the lacrimal glands [15, 37]. However, Ayyildiz et al. reported a significant relationship between lower Schirmer test scores and *Demodex* occurrence [13]. A probable reason was that the subjects in the study by Ayyildiz et al.were newly diagnosed DED patients with serious dry eye symptoms. Severe DED may have led to a decrease in ocular surface resistance, which could have resulted in an increase in *Demodex*. We think it is difficult to determine the causal relationship between them.

Though no significant differences were found between *Demodex* infestation and average LLT in both young and elderly patients, the average LLT was lower in *Demodex* positive individuals than in the other groups of young patients, while the average LLT was higher in the *Demodex* positive group than the other groups of elderly patients. The effect of *Demodex* presence on LLT may also be different in individuals of different ages. The relationship between tear film stability and lipid thickness is still controversial. Nevertheless, the exact thickness of the lipid layer that maintains tear film stability is still unknown, and the LLT should be assessed along with other dry eye parameters. Age is also an independent influential factor in LLT [38, 39]. Therefore, we considered that *Demodex* may affect the LLT value slightly. However, the relationship between *Demodex* infestation and LLT requires further investigation.

In this study, we compared all ocular surface and MG parameters between young and elderly patients in *Demodex* positive subjects. The MG dropout, lid margin abnormality, MG expression, plugging of MG orifices, and meibum quality were more serious and the FBUT was lower in the elderly than in the young patients. These results confirmed the findings of previous studies that a correlation between age and tear film and some MGD parameters. In the elderly, poor meibum could affect the plugging of MG ducts and orifices and reduce MG expression which may affect tear film stability [40, 41]. However, Li et al. demonstrated that MG loss was more serious in young patients with ocular demodicosis than in the elderly and suggested that *D. brevis* had a greater potential to cause severe MGD in young patients. However, its pathogenesis is unclear and requires further investigation. Further studies are also needed to investigate the role of *D. brevis* infestation in MGD [28].

According to our correlation analysis and linear regression analysis, the number of *Demodex* was significantly correlated with lid margin abnormality and meibum quality in young *Demodex* positive patients. However, in the elderly, the number of *Demodex* mites was correlated with the plugging of MG orifices. The results showed that after *Demodex* infestation, the number of mites was proportional to the severity of lid margin abnormality and meibum quality in young patients, and proportional to the plugging of MG orifices in the elderly.

There are several limitations to our study. No comparison was made between healthy people with and without *Demodex* infestation. We did not distinguish between *D. folliculorum* and *D. brevis*, so the role of *D. brevis* on MGD was unclear. Causal associations between *Demodex* infestation and different parameters were not confirmed. While eyelash sampling and microscopy are usually used to detect eyelash mites, *Demodex* accumulation in eyelash follicles may not be detected. Another important limitation of this study is the relatively small sample size and that no power calculations were performed prior to the study. Therefore, future studies with larger sample sizes and prospective designs are needed.

## Conclusion

Our study demonstrated that *Demodex* infestation was associated with MGD using the diagnostic criterion for *Demodex* infestation according to the expert consensus in China. The influence of *Demodex* infestation on the ocular surface and MG parameters of MGD was different in patients of different ages. In the young patients, dry eye symptoms, FBUT, CFS and MG expression did not change in *Demodex*-positive group compared with the suspicious-positive and negative group, but were more severe in *Demodex*-positive group of the elderly. In the young patients, meibum quality was worse in *Demodex*-positive group compared with the suspicious-positive and negative group, but not in *D*emodex-positive group of the elderly. MG dropout, plugging of MG orifices, lid margin abnormality were more serious in the *Demodex*-positive group compared with the suspicious-positive and negative group both in young and elderly patients. Therefore, the age or duration of *Demodex* infestation may play specific roles in *Demodex* infestation in MGD. Young patients with *Demodex* may be in the early stage of *Demodex* infestation. The pathogenesis of this relationship should be further investigated. It is necessary to pay more attention to the diagnosis and treatment of *Demodex* infestation in MGD. Because we were unable to determine whether MGD is the result of *Demodex* infestation, further studies are necessary to explore the causal relationship between *Demodex* infestation and MGD, especially the role of *D. brevis*. Our present findings provide guidance for early diagnosis and treatment of MGD patients with *Demodex* infestation. *Demodex* eradication could alleviate dry eye symptoms in elderly patients but not in young patients. However, *Demodex* eradication may alleviate other ocular symptoms such as itching in young patients. MG physical therapy is also necessary in young *Demodex* infestation patients.

## Data Availability

All data and materials were unpublished. For inquiries, please contact the corresponding author directly.

## Abbreviations

MGD: Meibomian gland dysfunction
MG: Meibomian gland
DED: Dry eye disease
OSDI: Ocular surface disease index
FBUT: Fluorescein tear film break-up time
CFS: Corneal fluorescein staining
SIT: Schirmer test I
LLT: Lipid layer thickness
